# Assessment of Strategies for Preserving Swine Viral RNA Targets in Diagnostic Specimens

**DOI:** 10.3390/microorganisms12020410

**Published:** 2024-02-18

**Authors:** Berenice Munguía-Ramírez, Luis Giménez-Lirola, Jeffrey Zimmerman

**Affiliations:** Veterinary Diagnostic and Production Animal Medicine Department, College of Veterinary Medicine, Iowa State University, Ames, IA 50011, USA; luisggl@iastate.edu (L.G.-L.); jjzimm@iastate.edu (J.Z.)

**Keywords:** swine viruses, viral RNA, RNA stability, diagnostic specimens, sample storage, molecular diagnostics

## Abstract

Successful downstream molecular analyses of viral ribonucleic acid (RNA) in diagnostic laboratories, e.g., reverse transcription-quantitative polymerase chain reaction (RT-qPCR) or next-generation sequencing, are dependent on the quality of the RNA in the specimen. In swine specimens, preserving the integrity of RNA requires proper sample handling at the time the sample is collected on the farm, during transport, and in the laboratory until RNA extraction is performed. Options for proper handling are limited to maintaining the cold chain or using commercial specimen storage matrices. Herein, we reviewed the refereed literature for evidence that commercial specimen storage matrices can play a role in preserving swine viral RNA in clinical specimens. Refereed publications were included if they compared RNA detection in matrix-treated vs. untreated samples. At present, the small number of refereed studies and the inconsistency in reported results preclude the routine use of commercial specimen storage matrices. For example, specimen storage matrices may be useful under specific circumstances, e.g., where it is mandatory to render the virus inactive. In a broader view, statistically sound side-by-side comparisons between specimens, viral RNA targets, and storage conditions are needed to establish if, when, and how commercial specimen storage matrices could be used in diagnostic medicine.

## 1. Introduction

Common swine ribonucleic acid (RNA) viruses, e.g., porcine reproductive and respiratory syndrome virus (PRRSV), porcine coronaviruses, swine influenza A virus, and others, are a threat to pig health and welfare. Measures taken to assess their presence on the farm require collecting specimens, e.g., serum, oral fluid, processing fluid, feces, environmental samples, semen, swabs, and tissues [1,2] for molecular testing, e.g., reverse transcription-quantitative polymerase chain reaction (RT-qPCR). In turn, these test results form the basis for decisions concerning their prevention and control. Regardless of sample status at the time of collection on the farm, RT-qPCR results reflect the quality and quantity of the target nucleic acid in the sample at the moment it is processed for testing in the laboratory [3,4]. However, between the time the sample is collected on the farm, packaged, shipped, and finally tested in the laboratory, it may have been exposed to handling conditions that adversely affect the RNA in the specimen and, therefore, the subsequent RT-qPCR test results. Notably, RNA is of more concern than deoxyribonucleic acid (DNA) in this regard because RNA molecules are susceptible to degradation via the hydrolysis of the 2′ and 3′ hydroxyl groups on their ribose residues. Herein, we provide an overview of RNA degradation and approaches to preserving swine viral RNA in diagnostic specimens.

## 2. RNA and Ribonucleases (RNases)

In vivo, RNA is continuously produced, which means that an active process of catabolism is necessary to eliminate defective or obsolescent molecules and maintain population equilibrium. For the most part, this process involves RNA-degrading enzymes, i.e., ribonucleases (RNases) [5,6,7]. RNases are hydrolytic enzymes that catalyze the cleavage of phosphodiester bonds to degrade RNA molecules into smaller fragments [8]. They are classified into two main groups with several types in each group: endoribonucleases, which cleave RNA molecules internally, and exoribonucleases, which digest RNA molecules from either the 3′ or 5′ end [5,9,10]. RNases are present in all cells and found in most secretions/excretions from living organisms. For that reason, RNases are ubiquitous in the laboratory environment, i.e., on human skin, laboratory glassware, metalware, and in laboratory working solutions [11,12,13,14]. RNases are heat-tolerant, stable over a wide range of pH, and resistant to many denaturing agents [15,16]. This justifies the requirement for working with samples in laminar flow hoods, wearing personal protective equipment, using RNase/DNase-free solutions, and treating labware and working solutions with potent RNase inhibitors such as diethyl pyrocarbonate (DEPC) or ribonucleoside-vanadyl complexes [13,17].

RNase A is the enzyme of main concern because it is ubiquitous [3,18]. A heat-resistant endoribonuclease, RNAse A, was first identified in 1920 [19], although it was not recognized as a ribonuclease until the 1930s [20,21,22,23]. RNAse A became commercially available in 1940 and, because of its thermostability and accessibility, was used extensively in protein studies in the 1950s and 1960s [24,25]. The predominant form of RNase A is non-glycosylated, but there are several RNase A glycoforms (RNases B, C, and D) [22,26]. All of these forms of RNase A contain four disulfide bonds (Cys26-Cys84, Cys40-Cys95, Cys58-Cys110, and Cys65-Cys72) that provide protection from denaturation; hence, RNase A is stable in the environment.

## 3. RNA Degradation and Diagnostic Testing

RNA includes both coding RNA or messenger RNA (mRNA) and non-coding RNAs, i.e., transfer RNA, ribosomal RNA, and small and long RNAs. Both coding and non-coding RNAs are recovered through the nucleic acid extraction procedure and targeted through polymerase chain reaction (PCR) primers and probes in the amplification step [27,28,29]. Hence, the responsibility of the veterinarian and the diagnostician is to protect the integrity of all RNA present in a diagnostic specimen. The “minimum information for publication of quantitative real-time PCR experiments (MIQE)” guidelines recognize sample storage as a key component in generating reliable and reproducible quantitative PCR (qPCR) data [30]. After diagnostic specimens are collected, and at any point during transport and storage, RNA degradation can occur through the action of ubiquitous, extracellular RNases that cleave RNA into fragments that are no longer recognizable by PCR primers and probes [5,31]. During cell lysis, RNases may be released from any specimen [12] but particularly from specimens with high RNase activity, e.g., pancreas, spleen, and lung [32,33,34]. Thus, extracellular RNases represent the primary threat to RNA integrity in molecular diagnostics [35,36].

Data on the effect of storage temperature on pathogen-specific RNA are sparse in the refereed literature, but the general effect is well established: RNA stability increases as temperature decreases; hence, the rule to keep samples at low temperatures, e.g., 4 °C, −20 °C, or −80 °C. A further complication is the fact that the temperature-dependent RNA decay rate varies among specimen types. For example, PRRSV RNA was relatively stable in serum at 4, 10, and 20 °C for 7 days, but a constant decline in PRRSV RNA concentration was observed over time in oral fluids and feces held at the same temperatures [37].

The need to preserve targets of interest in diagnostic specimens has been a topic of research since the 1920s [38,39]:

1. Freeze-drying (lyophilization). With the goal of finding a method to “*send active virus in small, sealed containers on sea voyages lasting over a month, and for long-term storage in the laboratory for several months without serious loss of virulence*,” in 1929, Sawyer reported that yellow fever virus could be preserved for over 155 days in “vacuum-dried” blood stored in sealed containers and refrigerated [39]. Lyophilization consists of freezing samples to immobilize water molecules and then placing them in a vacuum where the frozen water is vaporized, resulting in a dried specimen. This allows for prolonged storage of viruses in biological specimens that otherwise would be unstable in aqueous solutions [40]. In terms of nucleic acid stability, lyophilization is mostly used in vaccine production to preserve viral antigens and adjuvants to extend their shelf lives [41].
2. Viral transport medium (VTM). Attempts to improve virus storage have been described since the 1930s. Cook and Hudson [42] compared saline, water, human oral fluid, and serum (rabbit, sheep) and reported that sheep serum optimally preserved St. Louis encephalitis virus stored at 37 °C for 24 h. VTM consists of a mixture typically containing a buffered salt solution to maintain pH, antibiotics to prevent viral contamination, protein stabilizers (e.g., bovine serum albumin), and other additives intended to preserve viral integrity [43]. Although widely used for swab specimens, e.g., oral, nasopharyngeal, oropharyngeal, genital, and fecal swabs, VTM does not suit liquid specimens such as blood, serum, oral fluid, urine, etc. [44].
3. Untreated filter paper. The use of untreated filter paper (Guthrie Cards) for the transport and long-term storage of blood and urine began in the 1960s to detect phenylketonuria in infants [45]. Filter paper has long been used for storing and transporting fluid specimens, e.g., blood, saliva, and feces, intended for different assays, e.g., chemical assays, drug monitoring, nucleic acid or antigen detection, and serological markers for disease diagnostics. Nonetheless, filter paper is not typically used in routine viral diagnostics because eluting nucleic acids from specimens dried on the paper can lead to poor recovery and low nucleic acid yield [46].

Since accurate molecular testing is dependent on the quality and quantity of the nucleic acid material in the specimen, delivering intact RNA to the diagnostic laboratory is mandatory if reliable results are to be produced [3,47]. Although specimen stabilization technologies have been researched for over 100 years, the standard approach to RNA preservation remains the cold chain, i.e., chilling or freezing the specimen immediately after collection [37,48,49,50]. However, alternative approaches based on the use of commercial storage matrices emerged in the 1990s [51,52], and numerous commercial products are currently available (Table 1). The majority of these products are liquids to be combined with samples, but they also include solid surfaces onto which samples are spotted and dried. With some exceptions, these products are virucidal; thus, virus isolation or propagation is no longer an option.

Our objective was to review the use of currently available commercial storage matrices vis à vis viral RNA preservation in diagnostic specimens. The Google Scholar and National Library of Medicine PubMed search engines were queried using the Medical Subject Headings (MeSH) search term [RNA] with the Boolean operators AND [protectant OR protector OR protecting OR stabilizer OR stabilizing OR RNase inhibitor] AND [RNAlater OR RNasin OR DNA/RNA Shield OR RNAgard OR Monarch OR RNAprotect OR Oragene OR Aware Messenger OR Superase OR RNAsecure] AND [polymerase chain reaction OR RT-PCR]. The electronic search of the refereed literature produced 59 results in English language journals. Among these, 11 publications providing data on seven different storage matrices met the basic experimental design criteria, i.e., the researchers evaluated the capacity of a storage matrix to preserve viral RNA in diagnostic specimens (human or animal) on the basis of RT-PCR or RT-qPCR results and included an untreated control held under the same environmental conditions or stored frozen as a comparison. Notably, we identified at least nine commercial specimen storage protectants (Table 1) for which no refereed reports fulfilling our query parameters were discovered.

**RNAlater^TM^ Solution (Thermo Fisher Scientific, Inc., Waltham, MA, USA):** RNAlater^TM^ Solution is intended to preserve nucleic acids in tissue, cultured cells, bacteria, and yeast while maintaining viral infectivity [53]; “Invitrogen^TM^ RNAlater^TM^ Solution”, AM7020 datasheet, Thermo Fisher Scientific Baltics UAB: Lithuania, EU, January 2023 (https://www.thermofisher.com/order/catalog/product/AM7020, accessed on 25 October 2023). Among the five refereed publications that evaluated RNAlater^TM^ Solution and met the selection criteria, two described improved RNA detection in treated tissues vs. untreated controls. That is, RNAlater^TM^ Solution preserved classical swine fever virus (CSFV) RNA in spleen specimens held at 24 °C to 31 °C for 14 days when compared to control samples stored in glycerol/saline [54]. Based on daily testing using CSFV RT-PCR, the last positive control sample was on day 3 of exposure, whereas treated samples were consistently positive through day 14. Similarly, RNAlater^TM^ Solution preserved avian influenza virus in fecal homogenates exposed to ≤4 freeze–thaw cycles (x¯ Cq: 19.6) compared to samples without the storage solution (x¯ Cq: 25.5) [55]. In contrast, no difference in avian influenza virus Cq values was observed in cloacal swab samples stored in viral transport media vs. RNAlater^TM^ after 2 weeks of storage at either at 4 °C or “room temperature” [56]. Similarly, RNAlater^TM^ provided no benefit vs. viral transport medium in samples stored at 25 °C and tested for simian immunodeficiency virus (SIV) RNA at 1, 4, 8, or 12 weeks of storage [57]. In an additional study, the preservation of viral RNA using RNAlater^TM^ Solution was implied but not documented because untreated controls were not included in the design. Specifically, the RNA stability of hepatitis C virus (HCV) and human immunodeficiency virus (HIV) in plasma stored at 37 °C for up to 28 days in RNAlater^TM^ Solution was as good (x¯ = 1 × 10^5.64^ and 1 × 10^7.40^ molecules per mL, respectively) as flash frozen samples (x¯ = 1 × 10^5.59^ and 1 × 10^7.42^ molecules per mL, respectively) [58]. Indirectly supporting these data, other refereed publications reported that the concentration of HCV virus RNA declined in serum samples stored at “room temperature” for 5 days [59], and HIV RNA significantly decreased in plasma samples stored at 37 °C for 7 days [60].

**RNAprotect^®^ (Qiagen, Germantown, MD, USA):** RNAprotect^®^ reagents are intended for nucleic acid preservation in cells (RNAprotect^®^ Cell Reagent; Qiagen, Germantown, MD, USA), tissue (RNAprotect^®^ Tissue Reagent; Qiagen, Germantown, MD, USA), and oral fluid specimens (RNAprotect^®^ Saliva Reagent; Qiagen, Germantown, MD, USA). Although the formulation of these products is proprietary, they are known to include tetradecyltrimethylammonium oxalate (“RNAprotect Cell Reagent”, 600000002870 datasheet, QIAGEN Inc.: Maryland, USA, June 2023, https://www.qiagen.com/us/knowledge-and-support/product-and-technical-support/quality-and-safety-data/sds-search?l=US&q=600000002870%20, accessed on 25 October 2023; “RNAprotect^®^ Tissue Reagent”, 800000009992 datasheet, QIAGEN Inc.: Maryland, USA, September 2021, https://www.qiagen.com/us/knowledge-and-support/product-and-technical-support/quality-and-safety-data/sds-search?l=US&q=800000009992, accessed on 25 October 2023; “RNAprotect Saliva Reagent”, 600000002613 datasheet, QIAGEN Inc.: Maryland, USA, June 2023, https://www.qiagen.com/us/knowledge-and-support/product-and-technical-support/quality-and-safety-data/sds-search?l=US&q=600000002613, accessed on 25 October 2023), a cationic surfactant that precipitates RNA [61] and inactivates viruses [62]. No publications were found in which samples treated with RNAprotect^®^ Saliva Reagent were directly compared to untreated controls held under the same storage conditions. For example, one study on the stability of hepatitis E virus (HEV) in swine oral fluids reported a mean of 1 × 10^2.9^ genome copies per mL in samples stored at 37 °C for 24 h in RNAprotect^®^ Saliva Reagent vs. a mean of 1 × 10^5.0^ genome copies per mL in samples stored at −20 °C for 30 days but did not include any untreated (control) samples held 37 °C for 24 h for comparison [63].

One refereed publication that met the selection criteria tested two storage matrices, i.e., **Aware Messenger^TM^ (Calypte Biomedical Corporation, Portland, OR, USA)** and **Oragene^TM^ RNA (DNA Genotek, Ottawa, ON, Canada).** Aware Messenger^TM^ and Oragene^TM^ RNA consist of a collection swab and a capped tube containing a proprietary liquid transport matrix. No safety data sheet was found for Aware Messenger^TM^, but the safety data sheet for Oragene^TM^ (“ORAgene●RNA”, PD-MSDS-00006 datasheet, DNA Genotek Inc: Ontario, CA, July 2015, https://www.dnagenotek.com/us/pdf/PD-MSDS-00006.pdf, accessed on 25 October 2023) reported the inclusion of sodium dodecyl sulfate, glycine n, n’-trans-1, 2-cyclohexanediylbis n-(carboxymethyl)-hydrate, and lithium chloride in the medium. In a comparison of these two products, Decorte et al. [64] found no difference in PRRSV RNA concentration in oral fluid samples stored in Oragene^TM^ RNA vs. untreated samples stored at 4 °C and reported a lower concentration of PRRSV RNA in oral fluids stored in Aware Messenger^TM^ (1 × 10^3^ copies per mL) compared to untreated samples stored at 4 °C for 7 days (*p* = 0.001).

**PrimeStore^®^ MTM (Longhorn Vaccines and Diagnostics, San Antonio, TX, USA):** PrimeStore^®^ MTM is marketed as a transport medium for sputum, swab samples, blood-derived specimens, urine, feces, tissue, and environmental samples for viral and bacterial nucleic acids (Table 1). According to the safety data sheet, PrimeStore^®^ MTM contains guanidine thiocyanate, ethanol, trometamol, and sodium N-lauroylsarcosine (Longhorn Vaccines and Diagnostics LLC, “PrimeStore Molecular Transport Medium^®^ (MTM)”, LH-PSMTM-2-50 datasheet, EKF-diagnostic GmbH: Saxony-Anhalt, Germany, October 2020, https://www.ekfdiagnostics.com/res/Primestore%20MTM%20MSDS.pdf, accessed on 25 October 2023). The one refereed publication that fit the search criteria reported a slight loss of detectable influenza A (H1N1) RNA (∆Cq = 4) in human throat swabs stored in PrimeStore^®^ MTM at 38 °C and tested using RT-qPCR at 14 days of storage vs. no detection in samples stored in a commercial viral transport medium (BD Universal Viral Transport Medium, Baltimore, MD, USA) under identical conditions [65].

**TRIzol^®^ Reagent (Life Technologies, Carlsbad, CA, USA):** TRIzol^®^ Reagent, a solution based on phenol and guanidinium isothiocyanate, is considered the reference standard for RNA isolation [66]. TRIzol^®^ Reagent plus chloroform, a phase separation reagent, allows for the isolation of RNA, DNA, and protein fractions from biological samples [67]. TRIzol^®^ Reagent contains guanidinium isothiocyanate (“Trizol Reagent”, 15596026 datasheet, Life Technologies Corporation: Ontario, CA, May 2021, https://www.thermofisher.com/document-connect/document-connect.html?url=https://assets.thermofisher.com/TFS-Assets%2FLSG%2FSDS%2F15596026_MTR-NALT_EN.pdf, accessed on 25 October 23), a strong chaotropic denaturant that inactivates RNases present in the sample material [68]. Our search on the literature found one peer-reviewed publication fulfilling the selection parameters. Therein, Hofmann et al., 1999, reported that CSFV RNA in swine lymph nodes stored in TRIzol^®^ Reagent and held at 37 °C were detected for ≤4 weeks, whereas CSFV RNA in tissues stored in formaldehyde was detected for ≤1 week [69].

**Flinders Technology Associates (FTA)**^®^ **cards (Qiagen, Germantown, MD, USA):** FTA^®^ cards are cotton-based cellulose paper coated with a proprietary mixture of chemicals designed to denature proteins and lyse cells (“QIAcard FTA Indicating Classic,” 800000009302 datasheet, QIAGEN GmbH: Maryland, USA, February 2023, https://www.qiagen.com/us/knowledge-and-support/product-and-technical-support/quality-and-safety-data/sds-search?l=US&q=800000009302, accessed on 25 October 2023), as well as chelating agents and free-radical scavengers. FTA^®^ cards are intended for long-term, room-temperature storage of nucleic acids in blood, cultured cells, plasmids, and tissues. Tissue samples are collected as impression smears and liquid specimens are spotted on the cards and then dried. Keeler et al. [70] found similar avian influenza virus Cq values in cloacal and oropharyngeal swabs spotted on FTA^®^ cards and held at 23 °C for 7 days on the (mean Cq 22.8 and Cq 34.8, respectively) vs. samples collected in viral transport media and stored at −70 °C (mean Cq 26.8 and Cq 35.9, respectively). Linhares et al. [71] reported that the detection rate of PRRSV RNA in lung impression smears stored on FTA^®^ cards at 4 °C for 24 h was as good (11 positives among 62 samples) as fresh lung samples (11 positives among 62 samples), but the detection rate of PRRSV in serum samples spotted on FTA^®^ cards and held at 4 °C for 24 h was lower (40 positives among 74 samples) than fresh serum samples (45 positive among 74 samples). In oral fluids collected from pigs inoculated with PRRSV under experimental conditions [71], the overall detection of PRRSV RNA from 2 to 26 days post inoculation was lower in samples dried onto FTA^®^ cards and stored at 4 °C for 24 h (5 positives among 11 sample) compared to fresh oral fluids (11 positives among 11 samples). One notable complication in the assessment and comparison of FTA^®^ cards vis à vis RNA preservation is the fact that only a fraction of the card, i.e., 1.0, 1.2, 2.0, 3.0, or 6.0 mm, is punched and eluted for testing, effectively diluting the actual concentration of nucleic acids in the sample [70,71,72].

**Table 1:** Available commercial storage matrices and data on the efficacy of preserving viral RNA in diagnostic specimens ^a^.

Specimen Storage Matrix	Virucidal (Y/N)	Cost Per Sample ^b^	Indicated Specimen	Peer-Reviewed Data ^c^
RNAlater^TM^ Solution (Thermo Fisher Scientific, Waltham, MA, USA)	No	USD ~$2.43	Tissues, cultured cells, bacteria, yeast	CSFV in spleen [54]. AIV in fecal homogenates [55]. HCV and HIV in plasma [58]. AIV in cloacal swabs [56]. SIV in fecal samples [57].
RNAprotect^®^ Saliva Reagent (Qiagen, Germantown, MD, USA)	Yes	Price not listed	Oral fluid	HEV in oral fluid [63].
Aware Messenger^TM^ (Calypte Biomedical, Portland, OR, USA)	Not disclosed	Price not listed	Oral fluid	PRRSV in oral fluid [64].
Oragene^TM^ RNA (DNA Genotek, Ottawa, ON, Canada)	Yes	Price not listed	Oral fluid	PRRSV in oral fluid [64].
PrimeStore^®^ MTM (Longhorn Vaccines and Diagnostics, San Antonio, TX, USA)	Yes	USD ~$9.80	Sputum, swabs, blood, serum, urine, feces, tissue, environmental	IAV in throat swabs [65].
TRIzol^®^ Reagent (Life Technologies, Carlsbad, CA, USA)	Yes	USD ~$2.21	Cultured cells, tissue, bacteria, plant, yeast	CSFV in lymph nodes [69].
Flinders Technology Associates (FTA)^®^ cards (Qiagen)	Yes	USD ~$2.14	Blood, cultured cells, plasmids, tissue	AIV in cloacal and oropharyngeal swabs [70]. PRRSV in serum, oral fluid, and lungs [71].
DNA/RNA Shield^TM^ (Zymo Research, Irvine, CA, USA)	Yes	USD ~$0.60	Swabs, blood, feces, saliva, environmental, tissue, urine	No peer-reviewed publications
Monarch^®^ DNA/RNA Protection Reagent (New England Biolabs, Ipswich, MA, USA)	Not disclosed	USD ~$1.77	Tissue, swabs, oral fluid, blood, serum, feces	No peer-reviewed publications
RNAhold^®^ (Transgen Biotech, Beijing, China)	No	Price not listed	Cells and tissue	No peer-reviewed publications
RNAgard^®^ Blood System (Biomatrica, San Diego, CA, USA)	Yes	Price not listed	Whole blood	No peer-reviewed publications
PAXgene^®^ Blood RNA Tube (PreAnalytiX, Plymouth, UK)	Yes	USD ~$12.60	Whole blood	No peer-reviewed publications
Tempus^TM^ Blood RNA Tube (Applied Biosystems™, Burlington, ONT, Canada)	Yes	USD ~$9.62	Whole blood	No peer-reviewed publications
RNASound^TM^ Card (FortiusBio, Monterey Park, CA, USA)	Yes	USD ~$2.80	Serum, saliva, nasal fluid, environmental	No peer-reviewed publications
PowerProtect DNA/RNA (Qiagen)	Yes	USD ~$2.67	Feces	No peer-reviewed publications
RNAprotect^®^ Tissue Reagent (Qiagen)	Yes	USD ~$2.40	Tissue	No peer-reviewed publications

AIV: Avian influenza virus; CSFV: Classical swine fever virus; HCV: Hepatitis C virus; HEV: Hepatitis E virus; HIV: Human immunodeficiency virus; SIV: Simian immunodeficiency virus; IAV: Influenza A virus; PRRSV: Porcine reproductive and respiratory syndrome virus; ^a^ Sample storage matrices included in commercial nucleic acid extraction kits were not included unless peer-reviewed data meeting the selection criteria were found; ^b^ Prices listed on website as of January 2024: The cost per sample is based on the instructions for the use of each product; ^c^ Selection of the refereed literature based on the experimental design, i.e., the researchers evaluated the capacity of a storage matrix to preserve viral RNA in diagnostic specimens based on reverse-transcription PCR (RT-PCR) testing and included an untreated control for comparison.

**Non-commercial storage matrices.** There is some evidence that simple laboratory formulations could be protective of viral RNA [73,74]. Camacho-Sanchez et al. [73] described a “nucleic acid preservation buffer” based on ethylenediaminetetraacetic acid (EDTA), sodium citrate trisodium salt dihydrate, and ammonium sulfate. In a controlled comparison, rat (*Rattus rattus*) specimens (blood, liver, brain, muscle, and ear) stored in the “homemade” storage matrix at “room temperature” for 8 weeks yielded higher total RNA concentrations (ng/µL) than samples stored in RNAlater^TM^ under the same conditions. Similarly, Weidner et al. [74] reported that the storage of pharyngeal lavage samples containing severe acute respiratory syndrome coronavirus 2 (SARS-CoV-2) in a laboratory formulation containing 6 M guanidine-hydrochloride, 75 mM Tris-HCl, 30 mM EDTA-Na, and 1.5% (*v*/*v*) Triton X-100 resulted in significantly higher concentrations of RT-PCR-detectable SARS-CoV-2 over time (*p* < 0.0001; unpaired Student’s *t*-test) vs. samples stored in commercial media (Cobas^®^ PCR media, Roche Diagnostics, Rotkreuz, Switzerland) under identical conditions.

## 4. Considerations for Swine Veterinarians, Diagnosticians, and Researchers

The relevant question is, “For diagnostic samples intended for molecular testing, should specimens be placed in commercial storage matrices, or is cold storage sufficient?”. However, a review of the refereed literature revealed insufficient information to fully answer this question. The key issues include the following:

1. Sparseness of published data. There are relatively few reports in the peer-reviewed literature reporting the capacity of specimen storage matrices to preserve viral RNA in diagnostic samples over a range of storage conditions, e.g., storage temperature by time. The lack of refereed published data limits the ability of consumers to arrive at well-supported decisions regarding the use of commercial storage matrices.

2. Issues in experimental design. A lack of a uniform experimental design impedes the generalizability of the results, obfuscates their interpretation, and precludes side-by-side comparisons of storage matrices. Studies should always include untreated and treated samples exposed to identical conditions if accurate assessments of their effect on viral RNA preservation are to be achieved. Ideally, the comparisons should be reported in terms of the rate of inactivation over time, rather than the time over which the target was detected. This would require the evaluation of various temperatures and measurements at multiple time points to capture the temporal dynamics of nucleic acid stability over time. In contrast, most studies consisted of limited storage temperatures and measurements at relatively few time points.

3. Interpretation of divergent outcomes. Comparisons of treated vs. untreated samples reported both protection of viral RNA [54,55,58,65,69,70] and lack of protection. Lack of protection would include studies reporting similar RNA concentrations in both treated and untreated samples exposed to the same conditions [56,57] and studies reporting lower RNA concentrations in treated samples vs. untreated samples [64,71]. It is plausible that both outcomes are true and that the protective effect (where reported) may differ among specimen types and/or viruses, but there is insufficient data to detect a pattern of protection (or lack thereof).

4. The use of commercial specimen storage matrices may be appropriate in specific cases, e.g., when dealing with notifiable agents and virus inactivation is mandated. However, for routine use in the field, chilling or freezing may be sufficient to protect viral RNA in diagnostic specimens, e.g., storing serum at ≤20 °C and freezing oral fluid or fecal specimens at −80 °C [37]. Further, given that most commercial specimen storage matrices are virucidal, chilling or freezing samples provide the advantage of preserving virus viability, thereby providing for the possibility of further downstream analyses.

## 5. Conclusions

Delivering good-quality RNA to the laboratory is paramount if trustworthy molecular testing results are to be obtained. The objective of this study was to determine the role of commercial RNA storage matrices in this process. However, both the lack of controlled studies and weaknesses in experimental design in the published literature compromised our ability to make data-driven decisions regarding their use. Until stronger data in support of commercial storage matrices are available, maintaining the cold chain to preserve viral RNA in clinical specimens remains the best option for producers and veterinarians.

## Data Availability

Not applicable.
